# Recellularization of Bioengineered Scaffolds for Vascular Composite Allotransplantation

**DOI:** 10.3389/fsurg.2022.843677

**Published:** 2022-05-25

**Authors:** Aisha Adil, Michael Xu, Siba Haykal

**Affiliations:** ^1^ Latner Thoracic Surgery Laboratories, University Health Network, Toronto General Hospital, University of Toronto, Toronto, ON, Canada; ^2^ Institute of Medical Science, Temerty Faculty of Medicine, University of Toronto, Toronto, ON, Canada; ^3^ Division of General Surgery, Department of Surgery, University of Toronto, Toronto, ON, Canada; ^4^ Division of Plastic & Reconstructive Surgery, Department of Surgery, University of Toronto, Toronto, ON, Canada

**Keywords:** vascularized composite allotransplantation, tissue engineering, regenerative medicine, acellular scaffolds, recellularization

## Abstract

Traumatic injuries or cancer resection resulting in large volumetric soft tissue loss requires surgical reconstruction. Vascular composite allotransplantation (VCA) is an emerging reconstructive option that transfers multiple, complex tissues as a whole subunit from donor to recipient. Although promising, VCA is limited due to side effects of immunosuppression. Tissue-engineered scaffolds obtained by decellularization and recellularization hold great promise. Decellularization is a process that removes cellular materials while preserving the extracellular matrix architecture. Subsequent recellularization of these acellular scaffolds with recipient-specific cells can help circumvent adverse immune-mediated host responses and allow transplantation of allografts by reducing and possibly eliminating the need for immunosuppression. Recellularization of acellular tissue scaffolds is a technique that was first investigated and reported in whole organs. More recently, work has been performed to apply this technique to VCA. Additional work is needed to address barriers associated with tissue recellularization such as: cell type selection, cell distribution, and functionalization of the vasculature and musculature. These factors ultimately contribute to achieving tissue integration and viability following allotransplantation. The present work will review the current state-of-the-art in soft tissue scaffolds with specific emphasis on recellularization techniques. We will discuss biological and engineering process considerations, technical and scientific challenges, and the potential clinical impact of this technology to advance the field of VCA and reconstructive surgery.

## Contribution to the Field

Patients who experience traumatic injuries and severe tissue loss require surgical reconstruction. Large defects require alternative options whereby vascular composite allotransplantation involving the transfer of multiple tissues (e.g*.*, bone, skin, muscle, vessels, nerves) as a subunit is used in surgical reconstruction. The immunogenicity associated with VCA limits its clinical application. Decellularization and recellularization of these composite allografts is a potential solution to generate engineered VCA tissues. While work has been conducted to use decellularized tissues in VCA, the next frontier remaining is recellularizing these grafts with a view towards clinical translation. This review highlights the considerations and challenges required for establishing tissue-specific recellularization strategies including the biological and engineering parameters, current work and methods in the field, conceptual and technical challenges, and pertinent clinical applications of tissue engineering for VCA and reconstructive surgery. Foreseeably, the use of engineered VCA tissues may one day be applied to clinical practice to mitigate the issues of donor site morbidity and immunosuppression, thereby substantially improving patient outcomes and their quality of life.

## Introduction

Patients with traumatic injuries and large volumetric soft tissue loss require complex surgical reconstruction. Vascularized composite allotransplantation (VCA) involves the transfer of multiple, complex tissues as a whole composite subunit from immunologically compatible donors to recipients. Grafts used in VCA contain tissues such as the bone, skin, muscle, nerves, and vessels. Autologous reconstruction, the current standard, is limiting for patients with severe tissue and volumetric muscle loss due to limited donor tissue availability and donor site morbidity (1).

VCA grafts involve complex tissues such as the face, trachea, larynx, hand and leg extremities, abdominal wall, and genitourinary tissues. Many conditions do not have adequate reconstructive solutions for such complex tissues. Despite the promising outlook, long-term immunosuppression requirements limit the clinical application of VCA. Adverse effects include opportunistic infections, malignancies, end-organ toxicity, vascular impairment, variable graft survival, and lymphoproliferative conditions (1–3). Despite immunological compatibility between VCA donors and recipients, the immunogenicity of the different tissue types is also variable.

Tissue engineered scaffolds obtained through decellularization and recellularization are an alternative option to circumvent these limitations. Decellularization allows the removal of cellular and nuclear content while preserving the tissue extracellular matrix (ECM) architecture, resulting in an ECM scaffolds with retained tissue-specific composition and structural integrity (4). The methods for decellularization, process parameters, and evaluation of decellularized scaffolds has been evaluated in solid organs and individual tissues (5). The next frontier remains the recellularization of acellular composite allografts to a regenerative state for use in VCA. Recellularization conducted with recipient-specific cell populations using the native scaffold vascular network can create personalized allografts and help reduce the need for immunosuppression requirements.

**Figure 1:**
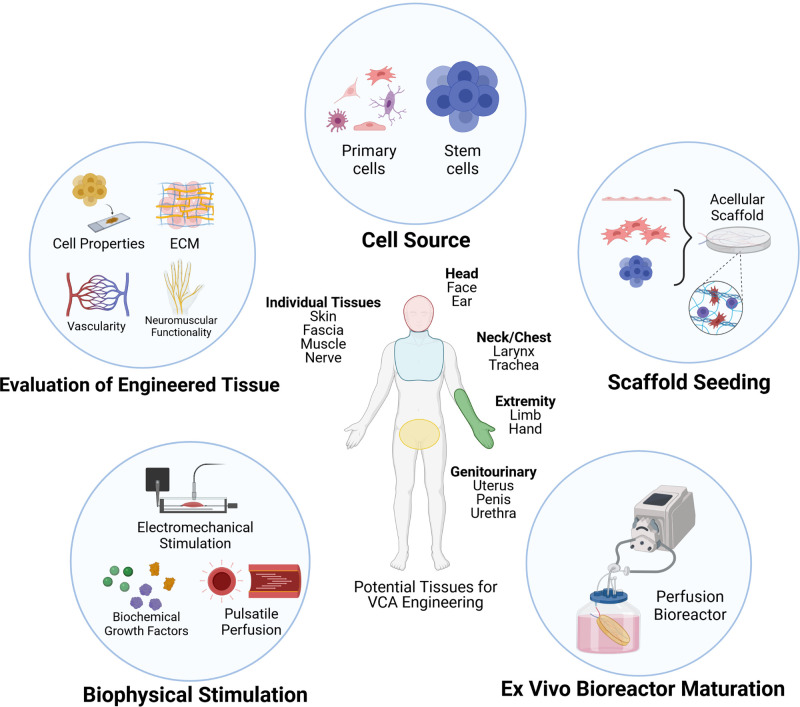
Approaches and considerations for recellularization of vascularized composite allografts (VCA). Figure created in ©BioRender—biorender.com (Toronto, ON).

The present review will focus on current state-of-the-art recellularization techniques in various soft-tissue scaffolds and examine several considerations pertinent to VCA tissue engineering (Figure 1). Factors such as cellular and matrix properties, recellularization approaches, and scaffold evaluation methods will be reviewed. The associated scientific and technical challenges, considerations for future directions, and clinical translatability of recellularized scaffolds will also be discussed.

## Cellular Properties for Recellularization

Fundamental to tissue engineering efforts is the selection and characteristics of cells used for a particular recellularization strategy. Given the complexity of VCA with its varied tissue compartments, a diverse population of cells is necessary for effective recellularization. Strategies to recellularize complex VCA tissues should include cells that comprise compartments such as the tissue parenchyma, stroma, and vasculature. To date, attempts to recellularize VCAs have incorporated a broad selection of cell types spanning from progenitor stem cells to terminally differentiated cells. The types of cells that have been used currently in VCA scaffold recellularization are reviewed below.

### Parenchymal and Stromal Cells

Parenchymal cells comprise the main cell population of a specific organ or tissue. As such, achieving sufficient recellularization of parenchyma and its supporting cells is essential to regenerating tissues that recapitulate normal physiological function. In cases of individual tissues such as muscle or nerve, parenchymal recellularization entails the use of cells such as myocytes and neurons, which serve as the fundamental units of their respective tissues. The recellularization of individual tissues can also include the use of progenitor cell populations, for example, myoblasts and neural stem cells, which offer greater potential for tissues regeneration. Indeed, previous *in vitro* work examining the co-culture of C2C12 myoblasts with PC12 neural stem cells showed increased myotube formation, neurite outgrowth, and improved muscle contractile force generation using both 2D and 3D methods to tissue engineer skeletal muscle constructs (6). These findings that are highly applicable in the regeneration of muscle with more complex VCA models.

In recellularizing complex composite tissues, additional parenchymal components need to be considered. Epithelial cells such as keratinocytes are needed in skin regeneration in order to recapitulate the epidermal barrier function as has been previously applied in the regeneration of a porcine fasciocutaneous flap (7). Recellularization of airway epithelium is critical to regenerating a functional upper respiratory tract capable of mucus production and mucociliary clearance as was done in the regeneration of laryngeal and tracheal tissues (8–10). Additionally, the inclusion of supporting stromal cells such as fibroblasts, chondrocytes, osteocytes, and adipocytes are important for their supportive roles as constituents of connective tissues. Fibroblasts, for example, represent the major constituent cell population found in connective tissues. Fibroblasts are involved in the production of ECM components to support tissue microarchitecture and mediate wound healing (11). Altogether, the diversity of cell types that constitute the parenchyma and stroma create a complex multicellular environment of cell-cell interactions that can maintain the overall viability of regenerated tissues.

### Cells of the Vascular System

Blood supply is of critical importance to recellularization of VCAs. The vascular system provides important oxygen and nutrient delivery via blood perfusion to support tissue viability and survival *in vivo* (12, 13). Specific to the integrity of the vascular system is the innermost lining composed of a monolayer of endothelial cells (ECs). Its chief function is to ensure a selective and non-thrombogenic barrier between vessel and tissue, as well as modulate inflammatory responses and vascular remodeling via angiogenesis (13–15). In addition to the endothelium, the surrounding mural cells, such as the pericytes and vascular smooth muscle cells, also play important roles in the vasculature. These mural cells interact closely with ECs and promote intercellular adherence and tight junctions that increase the endothelial barrier integrity (16). Pericytes, for example, can modulate endothelial growth, migration, and permeability *in vivo* (17–19). In tissue engineering, both ECs and the supporting mural cells constitute an important component of the vascular and microvascular niches (14), making them a critical component to recellularization efforts.

The use of endothelial cells for recellularizing acellular tissue scaffolds is guided by preceding work with whole organ engineering such as the decellularized kidney (20), liver (21), and lung (22, 23). While this work has been partly successful in recapitulating a viable endothelial layer with characteristic barrier function, work still remains to produce a complete endothelium absent of thrombogenesis upon *in vivo* transplantation in animal models (24–26). More recently, the concept of generating vascular chimerism in donor tissues was also proposed as a potential method to diminish immunogenic responses in organs. As the donor endothelium is one important mediator in transplant rejection, Cohen et al. hypothesized that selective replacement of endothelial cells in target organs and tissues, while keeping the remainder of the organ viable and functional may be an alternative approach to vascular regeneration that can reduce alloimmunity and increase survival of transplanted tissues (27, 28). Using tissues such as rat lung, kidney, and partial hindlimb models, the authors demonstrated the technical feasibility of selective de-endothelialization followed by *ex vivo* perfusion recellularization and culture of human placental-derived endothelial progenitors (EPCs) for 4 h at normothermic conditions to establish vascular chimerism (27).

### Stem Cells

#### Mesenchymal Stem Cells

Mesenchymal stem cells (MSCs) are a popular stem cell type commonly used in regenerative medicine and tissue engineering. Biologically, MSCs are stromal cells that exhibit a diversity of properties such as self-renewal, multi-lineage differentiation, immunomodulation and mediating cell-matrix interactions (26, 29–31). The use of MSCs is particularly relevant to vascular regeneration in VCA through their capacity to enhance angiogenesis and neovascularization by mechanisms that include direct trans-differentiation into vascular cell types and by the secretion of paracrine factors such as vascular endothelial growth factor and basic fibroblast growth factor (29, 32, 33). The immunomodulatory function of MSCs is also well known and can help promote constructive tissue remodeling and tissue survival following *in vivo* transplantation by suppression of host immune responses (34). Altogether, these properties, combined with their ease of acquisition in clinical settings make MSCs an attractive cell source for tissue regeneration and recellularization.

#### Induced Pluripotent Stem Cells

Recently, there has also been interest in the use of induced pluripotent stem cells (iPSC), which can be derived from the reprogramming of somatic cells with specific pluripotent factors that can reverse cell phenotype back to an “embryonic-like” state (35). Likewise, iPSCs are relatively easy to generate and have comparatively fewer ethical hurdles compared to embryonic stem cells, making them a good candidate for tissue engineering strategies (36). To date, the majority of work with iPSC scaffold recellularization has been performed in whole organ engineering, with examples of iPSC-derived endothelial cells seen in lung vascular (37, 38) and kidney vascular (39) engineering. However, there have been no reported attempts that utilize iPSCs in VCA recellularization, but such an approach may be an exciting future direction with great research and clinical potential.

## Extracellular Matrix Properties

The extracellular matrix (ECM) is a dynamic molecular network arranged in a tissue-specific ultrastructure that not only acts to structurally support the respective tissue but also regulate various cell functions such as cell migration, proliferation, differentiation, survival, and function to maintain cellular homeostasis (40). These activities are consequently variable across the unique composition and functional requirements of respective tissues (41, 42). Retaining such ECM properties provides tissue stability and a framework for recellularization to guide cell activity. Given the inherently complex structure and composition of the ECM, one that is not completely understood, it is difficult to synthesize and bioengineer such a construct. However, through decellularization, a native biologic ECM scaffold with retained ultrastructure and key proteins can be obtained. The significance of using an ECM scaffold is that it is a naturally occurring, highly conserved milieu of structural and molecular components important for cell growth and proliferation (43). Preserving the ECM can aid recellularization by providing biochemical cues to facilitate cellular attachment, viability, and tissue function (44).

The ECM comprises of structural glycoproteins such as fibronectin, glycosaminoglycans (GAGs), and laminin along with fibrous proteins such as collagen and elastin (4, 43). These key constituents have been of focus in previous recellularization studies. Further, the distribution and relative composition of these key constituents is tissue specific. This factor can explain the variability in the functional, structural, and mechanical properties observed across tissues (4, 45). This is significant because it introduces an added complexity to the decellularization and recellularization of composite tissues in particular. Methods of evaluating ECM constituents is also inconsistent and not defined by any criteria. Qualitative analyses include histological and immunohistochemistry (IHC)-based evaluations for most ECM components such as collagen, elastin, fibronectin, GAGs, and laminin whereas quantitative assays have been employed for collagen, elastin and GAG quantification (46). In evaluating recellularization through the lens of the ECM properties, one must consider the interdependency of the types of cells chosen for recellularization and their interactions with the ECM. The ECM is synthesized by the resident cell populations of respective tissues, which becomes more complex in composite tissue models (5). While extensive reviews and studies have been conducted on the effects of varying decellularization agents and their impacts on ECM proteins, the functions and roles of the listed ECM constituents in how they influence tissue recellularization has yet to be appreciated.

### Collagen

As the most abundant component of the ECM, collagen provides structural support in various isoforms with collagen type I primarily the most prominent isoform (4, 45). Tissues containing basement membranes show collagen IV located in basement membranes of vascular structures due to its ligand affinity with ECs whereas collagen VI serves as a connector between GAGs and functional proteins to collagen I. Other isoforms such as collagen III can be found in submucosal ECM. Given the variations of collagen types and their respective positions across differing parts of the ECM, the complexity required to recreate an ECM can be seen. While ECM scaffolds obtained through decellularization may retain collagen to varying extents, understanding the types of collagens and their respective locations in the ECM is critical to guide recellularization efforts. The relative amounts and locations of collagen types is crucial for cell growth to occur (45). When recellularizing either isolated or composite tissues, each tissue compartment could be analyzed for its respective collagen types to elucidate maintenance of collagen and how recellularization may have been supported by it. Collagen content has been consistently measured across recellularization studies using either histological or quantitative assay kits, with observably maintained or increased collagen content in most studies (2, 7, 47–53).

### Elastin

Another fibrous ECM protein is elastin, found in elastic fibers, which functions to recoil in tissues undergoing tensile force. In contributing to the ultrastructure and the mechanical properties of the ECM, examining relative retention of elastin after decellularization is significant for recellularization given its predominance in blood vessels, skin, cartilage, and adipose tissue (54). Further, while elastin has been investigated in various decellularization and recellularization studies, it is a component of a functional unit that is the elastic fiber. It exists in close conformation with microfibrillar glycoproteins, important for regulating and facilitating biomechanical properties of the ECM (54). Using Fastin elastin staining, elastin has been examined in most recellularization studies. Elastin was found to be preserved but decreased across studies of the porcine and human ears, porcine uterus and urethra, and vascularized skin flaps (7, 48, 50, 52, 55). In human ear decellularization, elastin expression was selectively decreased in the skin compartment. This particular finding highlights the need for preservation of elastin given its close relationship with collagen in maintaining cartilage mechanical properties. Preserving the ECM constituents that govern the tissue morphology and structure are critical to ensuring regeneration of tissue functionality, which in this case, involves the importance of elastin and collagen in cartilage for maintaining auricular function (9). Previous reports also indicate that the elasticity of an ECM contributes to both stem cell proliferation and differentiation. This is of particular interest for recellularization efforts with stem cells in particular and ensuring ECM scaffolds have retained elasticity properties (41).

### Fibronectin

Fibronectin is the second most abundant molecule in the ECM, after collagen, and is present in both tissue and soluble isoforms. Found in the basement membrane, interstitial tissue, and the submucosal membrane, fibronectin has a predominant position in the ECM. Fibronectin-rich ECM regions allow adhesion of multiple cell types along with facilitating cell migration, differentiation, and growth (4, 56). Fibronectin was found to be well preserved in recellularization of decellularized porcine ear, porcine uterus, porcine urethra, and human penile scaffolds based on immunofluorescence (48, 53).

### Glycosaminoglycans

GAGs are negatively charged, linear polysaccharide compounds often found covalently linked to core proteins of proteoglycans (57). These unbranched polysaccharides, GAGs, can be subdivided into two classes: sulfated GAGs (sGAGs) and non-sulfated GAGs. Sulfated GAGs include heparin sulfate, chondroitin sulfate, and keratin sulfate whereas non-sulfated GAGs include hyaluronic acid (4, 57). GAGs are involved in cell proliferation, differentiation, growth, and adhesion along with cytokine and growth factor binding (4, 46). For recellularization, retention of GAGs could be advantageous for retaining the tissue architecture as well as mechanical properties of a decellularized graft. GAGs have been detected in various studies using Alcian blue colorimetric assay kits (sGAG Dye Binding Assay, ALPCO, Salem, Northern Hampshire) or Blyscan Sulphated-GAG assay kit (Biocolor LTD, Carrickfergus, Northern Ireland), with the latter being the most common method. Although their identified need for providing biochemical cues and regulating cell function, GAGs are sensitive to preserve due to their position in the cell membrane. Reduction of GAGs can impact viscoelastic properties of grafts and the ability to retain water in the ECM (58). Many GAGs are located in cellular membranes that are often solubilized by decellularization agents (21, 44). In Duisit et al.’s investigation of the human ear scaffold, GAGs were significantly reduced in cartilage and skin tissues whereas in Jank et al.’s study on the rat forelimb, 40% of GAGs were retained (50, 51). Similar decreases in GAG content were reported for decellularized skin/adipose tissue flaps, human and rat face grafts, and penile scaffolds (2, 7, 47, 53). Lupon et al. suggests that this GAG reduction does not impact GAG-related functions in such decellularized ECM scaffolds due to the evident ability of recellularization, where cell attachment and proliferation can be observed. This is an interesting suggestion however it does not account for the variations in recellularization parameters across the studies of consideration. While cell attachment and proliferation may have occurred, other ECM activities GAGs influence such as growth factor binding and properties that affect the ECM’s gel-like properties require further investigation. It may also be considered that with recellularization, select cell types may secrete ECM factors that influence ECM composition and development. Further recellularization studies may consider these factors involving GAG quantification.

### Laminin

Found most commonly within basal lamina layers of tissues, laminin is a key adhesion protein that attaches to various cell types. It is found in numerous configurations, dependent on the peptide chains attached. Laminin is interconnected with collagen type IV through proteoglycans and glycoproteins (59). This protein has received close attention in scaffold vascularization due to its ability to organize and maintain vascular structures (43, 45). Mechanistic studies have also shown role of laminin in establishing and supporting endothelial barrier function as well as vascular permeability (59).

In considering the roles, functions, and evaluation methods for maintaining these ECM constituents, it must be highlighted that the preservation of such ECM components varies across methods of decellularization and sterilization procedures (60).

## Mechanical Properties

Contingent upon the ECM and tissue architecture are the mechanical properties of scaffolds. Mechanical testing is critical in considering the close relationship of mechanotransduction in influencing cell fate and differentiation, particularly for stem cells that differentiate based on matrix stiffness and elasticity (26, 61). Further, tissues’ distinct anatomic locations and function as part of a composite tissue dictate the biomechanical forces required in the design of bioreactor systems to mimic physiological conditions of respective tissues, and the types of mechanical tests used for evaluation of the scaffolds (12). Thus far, mechanical testing has only been conducted in decellularized human and porcine ear, rat forelimb, and porcine fasciocutaneous flap scaffolds (7, 48, 50, 51). Jank et al. performed the most extensive functional evaluations in the rat forelimb by assessing bone mineral density and content using peripheral dual-energy X-ray absorptiometry. Young’s modulus, bending stiffness and strength, and fracture energy were also evaluated. The range of motion in wrist and digit joints was well maintained post-decellularization (51). In porcine skin flap tissues, biaxial mechanical testing was also used to determine tissue stiffness changes. For muscle regeneration and functionality, myofiber morphometric measurement and isometric contractile force measurement was used. The latter technique is commonly employed for muscle function and muscle testing studies, a translatable component for recellularization studies focusing on muscle regeneration. Other examples include ball burst tests and tensile strength tests used in decellularized human ear scaffolds, and Young’s modulus measurements used in decellularized porcine ear scaffolds (48, 50).

No mechanical analyses post-recellularization were conducted in any of these studies. This may be attributable to the challenges in regenerating tissues to their functional states in composite tissues after recellularization. Many of these studies achieved cell engraftment and function after cell seeding but did not pursue recellularization strategies to promote tissue function regeneration which would otherwise require long-term culturing, implementation of biophysical stimuli during recellularization, and considerations for tissue-specific mechanical properties. For example, measurements for shear stress may be considered for blood vessels post-recellularization given that endothelial cells change phenotypes when shear stress is induced (12). The importance of analyzing mechanical properties post-recellularization cannot be understated as the seeded cells sense and rely on the surrounding mechanical environment and thus, heavily impact composite tissue recellularization outcomes.

## Recellularization Bioreactor Systems

*Ex vivo* bioreactor systems have been commonly employed in tissue recellularization studies, with influence drawn from previous solid organ-based de- and recellularization work. The design of bioreactor systems is often tailored to mimic *in vivo* physiological conditions that can support the tissue for at least 1–3 weeks (62). Key features for a bioreactor system include the ability to tailor flow rates, ability to monitor culture condition parameters (e.g., pH, oxygen, metabolites), leak-proof and sterilizable components, easy assembly, and ease in inserting/retrieving scaffolds (63). Commonly employed bioreactor systems for VCA tissue recellularization have included spinner flasks and perfusion bioreactors, with latter as most common. Considerations for bioreactor environments include tailored cell culture conditions and provision of biophysical stimuli based on the tissue of interest’s composition and function.

Recellularization is heavily reliant on culture conditions to support cell survival and growth within scaffolds with considerations required for different cell types, cell medium composition, respective growth factors and supplements, and cell seeding densities (12). Tailoring recellularization strategies to control the stages of proliferation, differentiation, and subsequently, maturation of cells is also advantageous in regenerating tissues. Juhas et al. identified growth factors that can help promote myogenic proliferation but inhibit differentiation including basic fibroblast growth factor and epidermal growth factor whereas for myotube formation, insulin-like growth factor-1 and transforming growth factor-β1 were suggested (64). Many of these angiogenic factors are required for other cell types, introducing the need for tailoring cell medium composition to support co-cultures. Moser et al.’s recellularization attempts with a canine larynx involved significant characterization of cells in different medium conditions and supplements to determine a universal cell culture medium conducive to the growth and differentiation capabilities of primary human myoblasts, human airway basal cells, and human umbilical vein endothelial cells (HUVECs) (8). Hence, regeneration of composite tissues has an interdependency on culturing and maturation strategies to help accelerate tissue maturation and eventually tissue functionalization.

Biophysical stimuli are also a significant design consideration for bioreactor environments, requiring the inclusion of chemical, electrical and/or physical stimulation to support a growing tissue. For example, a decellularized muscle will require progenitor muscle cells to help form muscle however complete skeletal muscle development is reliant on functional innervation to mimic neuronal activity typically required in native muscle (65). Jank et al. employed electrical stimulation by immersing carbon rods in the bioreactor environment for the rat forelimb and established an electrical field stimulation to promote muscle cell alignment and differentiation (51). Previous work on engineered skeletal muscle and application of electrical stimulation has substantial evidence to suggest the positive influence of electrical stimulation on muscle cell differentiation, alignment, and ability to promote myofiber arrangements (65, 66). This is significant for functional tissue development, as maturation of cell types to the matured phenotype is required for proper tissue development.

## Recellularization Methods of VCA Tissues

Examples of recellularization of both individual and composite tissues that are relevant to VCA are discussed below and are summarized in Table 1. Individual tissues focus primarily on muscle, and nerve tissues while composite tissues focus on the following: fasciocutaneous flaps, face, ear, larynx, trachea, limb, and genitourinary tissues.

**Table 1:** Summary of vascularized composite allograft recellularization studies across individual and composite tissue models.

Scaffold, Species	Decellularization Method (method, reagent, duration)	Sterilization (method, reagent, duration)	Cell Types	Method of Seeding	Cell Number	Machine Perfusion (Y/N), Flow Rate	Duration	Transplantation	Methods of Analyses	Main Conclusions
Superficial epigastric skin flap, Rat (48)	Not decellularized	N/A	MAPCs, BMSCs, ADSCs	Manual perfusion	N/A	Y, <0.2 mL/min	≤24 h	Y	H&E, IHC, FISH, NBT viability staining, BrdU, luciferase detection	Intact vascular network, cell engraftment and active cell proliferation observed
Skin/adipose flap, Rat (3)	(1) Freeze-thaw at −80 °C, 3 cycles. (2) Perfusion with NaCl (0.5 M and 1 M) for 4 h each and repeated once, 0.25% trypsin/EDTA for 2 h at 37 °C, agitation with isopropanol overnight, and 1% Triton for 48 h	70% ethanol and rinsed in PBS	HUVECs, HUVECs + hADSCs	Manual Perfusion, Injection	2.5 × 10^5^ HUVECs perfused, 1 × 10^6^ hADSCs + 2.5 × 10^5^ HUVECs injected	N	7 days	Y	H&E, Masson’s, IHC, DAPI staining, micro-CT, SEM, live/dead staining	Native ECM and biochemical properties maintained, HUVECs showed proliferation and endothelialization, M1 and M2 macrophage infiltration post-transplantation, thrombosis noted following transplantation
Fasciocutaneous groin flap, Pig (7)	Perfusion with 1% SDS for 10 days, perfusion with 1% Triton X-100 for 1 day	N/A	HUVECs	Gravity perfusion, static culture for 2 h	40 × 10^6^ HUVECs	N	5 days	Y	H&E, IHC, SEM, biaxial tensile strength, DNA & sGAG quantification, biotin assay	Biochemical and histological decellularization confirmed, native tensile strength maintained, no venous outflow post-transplantation, limited re-endothelialization observed
Intercostal Nerve, Pig (67)	(1) Freeze-thaw in liquid nitrogen for 30 min, 3 cycles. (2) Digested in 0.25% trypsin for 30 min, incubation and agitation with 0.1% SDS and 3% Triton X-100 for 12 h, agitation with DNAse/RNAse	0.1% paracetic acid for 3 h, PBS wash for 3 h	Neural differentiated ADSCs, Schwann cells	Injection	5 × 10^6^ cells/mL	N	4 days	Y	H&E, IHC, SEM, WB, MTT assay, electrophysiology	ECM structure and scaffold properties maintained, cell engraftment and neural tropic factor expression detected, neural cells longitudinally aligned post-recellularization, lack of reinnervation with recellularization via electrophysiology
Sciatic Nerve, Rat (68)	Incubation with 3% Triton X-100 overnight, incubation and agitation in 4% SDC for 24 h	Irradiation with Co^60^ for 12 h	Schwann cells differentiated from ADSCs and BMSCs	Injection	5 × 10^5^ ADSC-SC, BMSC-SC, authentic Schwann cells	N	2 weeks and 12 weeks	Y	Toludine blue staining, TEM, IF, von Frey hair sensitivity test, muscle contraction	Recellularized grafts showed regeneration capacities and presence of myelineated fibers, limited sensory function post-recellularization, less muscle atrophy post-recellularization
Sciatic Nerve, Pig (69)	Perfusion of 1% SDS for 50 h, perfusion of 1% Triton X-100 for 5 h, DNAse treatment, perfusion MgCl for 12 h	N/A	wtPAEC	Manual perfusion, split in 4 consecutive injections	1 × 10^7^	Y, 0.5 mL/min–2.0 mL/min	7 days	N	H&E, GAG and DNA quantification, DAPI staining, micro-CT, multiplex assays, IHC,	ECM and biochemical properties maintained, DNA content reduction, vessel-like structures observed after 7 days recellularization, cell engraftment in only proximal portions of of graft
Rectus Abdominus, Pig (3)	Perfusion of 0.02% trypsin/0.05% EGTA for 1.75 h (arterial) and 0.25 h (venous), 0.1% SDS perfusion for 12 h (11 h via. artery and 1 h via. vein), perfusion of 0.1% Triton X-100 for 12 h (11 h via. artery and 1 via. vein)	0.1% paracetic acid/4% ethanol for 2 h and perfusion of deionized water for 7 days	C2C12 myoblasts	Static seeding	0.5 × 10^5^	N	24 h	N	H&E, Masson’s, SEM, sGAG quantification, tensile strength test, growth factor analysis	Seeded myoblasts expressed mature muscle markers 24 h post-seeding, decellularized matrices maintained ECM components and tissue ultrastructure
Hemifacial graft, Rat (35)	Protocol I: Perfusion with 1% SDS for 100 h, rinse with PBS for 24 h. Protocol II: Mechanical agitation using 1% SDS for 140 h and PBS for 72 h.	Agitation with 0.1% paracetic acid overnight, rinse with PBS.	hADSC	Static seeding	5 × 10^5^	N	7 days	N	H&E, IHC, DNA quantification, microCT	Both mechanical agitation and perfusion decellularization methods were successful. Viable and engrafted cells found on dermal and internal portions of recellularized scaffold. Some cell migration observed.
Face, Human (2)	Perfusion with adenosine, 1% SDS, 1% Triton X-100, and PBS. Agitation overnight and subsequent perfusion with 2-propanol for 12 h for defatting. Overnight agitation in 2-propanol. DNAse treatment.	0.1% paracetic acid	NIH-3T3, C2C12 myoblast cells, HAECs	Perfusion, static seeding, injection	50 × 10^4^ NIH-3T3 + 4 × 10^6^ C2C12, 70 × 10^6^ C2C12 only, 25 × 10^6^ HAECs	Y, 2 −4 mL/min (C2C12), 1 mL/min (HAECs)	4 h, 48 h, 14 days	N	H&E, IHC, ECM/DNA quantification, cell function assays	Successful decellularization, preserved tissue structure. Seeded cells showed viability and engraftment upon recellularization. Cells were well distributed in scaffolds.
Ear, Pig (28)	Perfusion with 1% SDS, rinse with 1% Triton X-100 and PBS. DNAse treatment.	N/A	BMSCs, NIH-3T3	Manual perfusion, injection	16 × 10^6^ BMSCs (manual perfusion), 70 × 10^6^ NIH-3T3 cells (injection)	Y, 2–4 mL/min (BMSCs), 1–4 mL/min (NIH-3T3)	14 days (BMSCs), 10 days (NIH-3T3)	Y	H&E, IHC, SEM, mechanical testing, DNA/ECM quantification, AngioCT, Sudan lipid staining	ECM content and tissue structure maintained post-decellularization. NIH-3T3-seeded grafts showed high cell engraftment and cell clusters in both extra- and intravascular compartments. BMSC-seeded grafts showed cell migration throughout scaffold and increased lipid content.
Ear, Human (51)	Perfusion with 1% SDS for 88 h, 1% Triton X-100 for 25 h, PBS for 38 h, 2-propanol for 4 h. Agitation overnight in 2-propanol. DNAse treatment.	Washed in 0.1% paracetic acid overnight, rinsed in distilled water, washed with PBS.	Rat ADSCs, HAECs	Perfusion, static seeding	3.6 × 10^4^ (rADSCs), 30 × 10^6^ (HAECs)	Y, 2–6 mL/min (HAECs)	48 h (HAECs), 2 weeks (rADSCs)	N	H&E, Masson’s, Alcian Blue, IHC, lipid droplet evaluation, SEM, MTT, ECM protein/DNA quantifcation, live/dead staining, mechanical testing, cytokine quantification,	Successful decellularization, preservation of ECM content and structure, reduced DNA content. Cell engraftment and viability observed across both cell types and both cell seeding methods.
Larynx, Rabbit (70)	Perfusion with 1% SDS for 16 h, perfusion with 1% Triton X-100 for 30 min, perfusion with antibiotic-containing PBS for 48 h.	Placed in DMEM with 15% FBS	BMSCs	Injection	N/A	N	24 h	N	H&E, IHC, SEM	Maintained ECM and tissue content post-decellularization, vessel-like structures observed 8 weeks post-transplantation, recellularization concentrated at injection sites only, partial formation of muscle bundles observed
Larynx, Canine (8)	Perfusion with 1% SDS for 5 days, perfusion with 1% Triton X-100 for 6 h, wash with PBS for 4 days	N/A	HUVECs, hBCs, hMBs	Perfusion, injection	80 × 10^6^ HUVECs, 2 × 10^6^ hMBs, 700,000 cells/cm^2^ hBCs	Y, 1–2 mL/min (HUVECs only)	8 days (HUVECs), 5 days (hBCs), 5 days (hMBs)	N	H&E, IHC, GAG/DNA quantification, CT scan, biomechanical testing, cell function assays	Maintained ECM and tissue structure post-decellularization, endothelial lining observed in both artery and vein, hHBCs were engrafted scucessfully, cell proliferation and viability detected in laryngeal muscle post-recellularization
Trachea, Pig (9)	Protocol I: Immersion in 3% Triton X-100 for 48 h with mechanical agitation. Protocol II: Stirred in hypotonic solutions of 10 mM Tris HCl, 5 mM EDTA, 1% Triton X-100, PefablocPlus™ (protease inhibitor), antibiotics/antimycotics at 4 °C for 24–48 h	Protocol I: wash with 0.1% peracetic acid, 4% ethanol, and 96% dionized water for 2 h with mechanical shaker. Protocol II: Wash 4 times in sterile distilled water.	BMSCs, TECs	Static, Perfusion	6 × 10^7^ (for static and perfusion, each)	Y, 1.5 mL/min	72 h	Y	H&E, IHC, cell toxicity and proliferation, cell labeling	Perfusion bioreactor non-toxic, perfusion-seeded grafts showed increased cell counts, higher homogeneity scores, labeled TEC found on luminal surface post-recellularization, labeled BMSC found on external surface only post-recellularization. Post-transplantation, revascularization and re-epithelialization observed at distal segments of graft
Trachea, Pig (10)	Immersion in 1% SDS while scaffolds rotate for 3 h, immersion in 1% Triton X-100, immersion in PBS for 30 min	N/A	BEAS-2Bs	Injection	1 × 10^6^	Y, 1/5 mL/min	7 days	N	H&E, Masson’s, Alcian Blue staining, IHC, tensile test, SEM, live/dead assay, sGAG quantification, cell metabolism	Successful de-epithelialization with preservation of tissue ultrastructure, ECM content. Viscoelasticity maintained post-decellularization. Grafts were able to support cell engraftment and viability until 24 h *in vitro.* Viable epithelium observed in bioreactor-based recellularization.
Forelimb, Rat (66)	Perfusion with 1% SDS for 50 h, perfusion with 1% Triton X-100 for 1 h	Perfusion with antibiotic-containing PBS for 124 h	HUVECs, C2C12 myoblasts, MEFs	Gravity perfusion, injection	5 × 10^6^ HUVECs, 10 × 10^6^ C2C12 myoblasts, 0.5 × 10^6^ MEFs	Y, 1 mL/min	21 days	Y	H&E, IHC, mechanical bone testing, tensile strength test, isometric muscle contraction	Cell engraftment observed, muscle-like tissue formation with injected myoblasts, ECM properties maintained, reduced DNA content, venous return not reported, flexion of joints observed upon electrical stimulation post-recellularization
Penis, Human (54)	Perfusion with 1% SDS for 14 days with simultaneous slow mechanical agitation	N/A	SVF	Injection	1 × 10^6^	N	1 day, 28 days	N	H&E, DAPI, IHC, SEM, DNA quantification	ECM content and tissue structure maintained post-decellularization. High density of viable cells observed 28 days post-recellularization throughout graft. Some differentiated smooth muscle cells observed.
Corpus Cavernosum, Rabbit (71)	Incubation with 2% Triton X-100 and 0.1% ammonium hydroxide for 10 days with simultaneous mechanical agitation.	N/A	Muscle-derived stem cells	Static seeding	30 × 10^6^ cells/mL	N	5 days	Y	H&E, IHC, SEM, WB	Tissue ultrastructure maintained, cell engraftment and proliferation observed until 5 days post-recellularization, transplanted acellular tissues showed neovascularization and cell proliferation
Corpus Cavernosum, Rabbit (72)	Incubation with 2% Triton X-100 and 0.1% ammonium hydroxide for 10 days with simultaneous mechanical agitation.		VEGF-transfected muscle-derived stem cells	Static seeding	30 × 10^6^ cells/mL	N	3 days	Y	H&E, IHC, SEM, WB	VEGF-transfected stem cells showed better cell engraftment and cell growth post-recellularization. Stem cell differentiation and endothelial markers showed expression at different timepoints.
Urethra, Pig (53)	Perfusion with either: 1% Triton X-100 and 0.5% ammonium hydroxide, 0.1% SDS, 0.5% SDS, and 1% SDS for 5 days. Direction of perfusion reversed after 48 h. Perfusion with distilled water for ≥ 12 h.	Washed with antibiotic and antimycotic-containing PBS	Human MPCs, hBMSC, SVF, L929 Fibroblasts	Static seeding	2 × 10^6^ each	N	14 days	N	H&E, IHC, DNA/sGAG/ECM protein quantification, fiber formation assay	Only 1% and 0.5% SDS showed decellularization. ECM structure and protein content maintained. Cell engraftment successful, MPCs differentiated into multinucleated fibers, BMSCs and L929 fibroblasts homogenously adhered. Only L929 proliferated after 14 days post-recellularization.
Uterus, Rat (73)	Perfusion with PBS overnight, 0.01% SDS perfusion for 72 h, perfusion with 0.1% SDS for 24 h, perfusion with 1% Triton X-100 for 30 min.	Antibiotic and antimycotic-containing PBS for 1 week	Neonatal and adult rat uterine cells, rat MSCs	Injection	5.1 × 10^7^ neonatal rat uterine cells, 2.7 × 10^7^ adult rat neonatal cells, 1 × 10^6^ rat MSCs	Y, 15 mL/min	3, 6, 10 days	N	H&E, Masson’s, TEM, IHC	Uterine cell engraftment observed, partial cell distribution observed after 3 days post-seeding, vessel-like structure proteins detectable but not robust, atrophic tissue observed 6 days post-seeding
Uterus, Pig (50)	Protocol I: Freeze-thaw, 2 cycles. Perfusion with 0.1% SDS for 18 h, perfusion with 1% Triton X-100 for 30 min. Protocol II: Perfusion with 0.1% SDS for 18 h, perfusion with 1% Triton X-100 for 30 min. Repeated protocol twice.	UV exposure for 2 h	Mixture of stromal and epithelial stem cells (human endometrial derived)	Injection	5 × 10^4^	N	3, 6, 9 days	N	H&E, IHC, SEM, Alcian blue staining, Vascular corrosion cast, DNA quantification	Morphology and ultrastructure maintained, most ECM proteins maintained, GAG content reduced, DNA content reduced, contraction of recellularized tissue observed 3–4 days post-seeding
Uterus, Ovine (56)	Protocol I: Perfusion of 0.5% SDS for 8 h, perfusion of PBS overnight, DNAse treatment. Protocol II: Perfusion of 2% SDC for 8 h, perfusion of PBS overnight, DNase I treatment. Protocol III: Perfusion of 2% SDC for 4 h, distilled water for 6 h, 1% Triton X-100 for 12 h, distilled water for 36 h, DNase I treatment at 37 °C. All tissues frozen in distilled water at −20 °C	Perfusion of 0.1% peracetic acid in a 0.9% NaCl, 1 h. Followed by perfusion of sterile PBS, 48 h.	Sheep feotal BMSCs	Injection	1 × 10^6^ and 1 × 10^7^ separately	N	3 days and 14 days	N	H&E, IHC, Masson’s, Alcian Blue, SEM, DNA/protein/ECM quantification, MTT assay, mechanical test	ECM content preserved post-decellularization, cell engraftment and viability observed until 14 days, cells remained in superficial layers of tissue and localized to site of injection with no difference between two cell density conditions

*Abbreviations: ADSC, adipose-derived stem cell; BMSC, bone marrow-derived stem cell; DMEM, Dulbecco’s modified Eagle medium; ECM, extracellular matrix; HAEC, human aortic endothelial cell; hBCs, human airway basal cells; HCl, hydrogen chloride; hMBs, (primary) human myoblasts; HUVEC, human umbilical vein endothelial cell; IF, immunofluorescence; IHC, immunohistochemistry; MAPC, multipotent adult progenitor cell; MEF, mouse embryonic fibroblasts; MPC, muscle progenitor cell; NaCl, sodium chloride; SEM, standard electron microscopy; sGAG, sulfated glycosaminoglycans; SDC, sodium deoxycholate; SDS, sodium dodecyl sulfate; SVF, stromal vascular fraction cells; TEM, transmission electron microscopy; VEGF, vascular endothelial growth factor; WB, western blot; wtPAEC, wild type porcine aortic endothelial cells.*

### Individual Tissues

#### Muscle

Zhang et al. explored perfusion decellularization and recellularization of skeletal muscle obtained from porcine rectus abdominus. Decellularization was conducted through both antegrade and retrograde perfusion using 0.02% trypsin/0.05% EGTA, 0.1% sodium dodecyl sulfate (SDS) and 1% Triton X-100 each through the inferior epigastric vessels, with more perfusion time in retrograde perfusion than antegrade. For recellularization, C2C12 mouse myoblasts were statically seeded onto pepsin-digested decellularized matrices in 24-well plates. Seeded myoblasts showed higher expression of myosin-heavy chain (marker of matured muscle) after 1 day post-differentiation, higher fiber diameter, and higher fusion index relative to the control tissue, small intestinal submucosa ECM. While perfusion recellularization was not pursued, the seeded cells indicated an ability for differentiation and showed evidence for a preserved ECM to allow cell growth and maturation. However, the stages of myoblast cell proliferation and differentiation were not monitored long-term nor were details of how differentiation was induced given. Further, no muscle stimulation nor characterization of muscle contraction of the differentiated cells was conducted. Separately, Zhang et al. attempted to use decellularized grafts to treat partial thickness abdominal wall defects in rats. Eight weeks after surgery, long ovoid nuclei in nerve bundle membranes could be detected, similar to Schwann cell nuclei morphology. As well, some organized neo-muscle fibers could also be observed along the endomysium (3).

#### Nerve

For nerve injuries, multiple surgical reconstructive options are available such as autologous nerve grafting, bone excisions to shorten large nerve gaps, and nerve transfers. However, many limitations exist such as limited graft availability, scarring, donor site morbidity, challenges in procuring relevant nerve size and morphologies, and delayed or impaired reinnervation post-surgery. Using nerve allografts to fill nerve gaps between the proximal and distal ends of the defected nerve is an alternative approach. Many natural and synthetic-based biomaterials have been used for preparing nerve conduits to bridge nerve gaps. Of these, some nerve conduits are commercially available for nerve gap repair although its application in long nerve gap (>3 cm) repair has had mixed results (67, 74). Further, an additional challenge is ensuring conduits contain regenerative capacity. Some studies have attempted applying methods of de- and recellularization in nerve grafts.

Using neural-derived ADSCs, Zhang et al. proposed recellularization methods in a rat intercostal nerve model. Segments of intercostal nerves procured from pigs were subjected to a multi-step decellularization process involving freeze-thaw cycles, 0.25% trypsin exposure, 0.1% SDS/3% Triton X-100 incubation, with agitation. Notably, authors used 1-ethyl-3 (3-dimethylaminopropyl) carbodiimide (EDC) to facilitate crosslinking. This is a method employed in various solid organ decellularization studies, used to improve mechanical properties of decellularized scaffolds (69). The ECM structure and porosity, and some growth factors were preserved upon decellularization. Recellularization involved mixing neural differentiated ADSCs with collagen gel and injecting the cell suspension on proximal and distal ends of the nerve. The nerves were incubated for coagulation before being cultured in medium for 4 days. Some recellularized nerves were used to bridge sciatic nerve gaps in rats. Cells were labeled with PKH-26 and authors traced the neural cells *in vivo* post-transplantation, revealing longitudinally aligned neural cells and expression of neural tropic factors. Seven days post-transplantation, authors also reported glial cell characteristics in the neural differentiated ADSCs (74).

Wang et al. proposed methods of recellularization with Schwann cells in Wistar and Sprague-Dawley rats. Autologous Schwann cells are difficult to expand and obtain hence authors identified the need for alternative sources of Schwann cells for recellularization. BMSC and ADSCs isolated from rat femur, tibias, and subcutaneous back fat tissues were differentiated into Schwann cells. Sciatic nerves measuring 20 mm were decellularized using a previously established protocol with 3% Triton X-100 overnight followed by 4% sodium deoxycholate (SDC) with agitation for an additional 24 h (75, 76). For recellularization, 5 treatment groups were tested. Left sciatic nerves of all rats were exposed and used to create a 15 mm gap. While control group animals had an immediate nerve repair using the transected nerves, the other 4 treatment groups involved nerve repair using acellular nerve grafts whereby 5 × 10^5^ Schwann cells were injected through both proximal and distal ends of the nerve grafts. Authentic Schwann cells, BMSC-derived and ADSC-derived Schwann cells as well as cells suspended in DMEM were used in the remaining 4 treatment groups. Analyses were conducted at 2-week and 12-week intervals. BMSC and ADSC Schwann cells independently showed regeneration and functional recovery of the sciatic nerves whereby recellularization with either cell type was comparable across autografting with Schwann cells alone and better than acellular nerve autografting. Authors identified alternative Schwann cell sources and the enhanced regenerative capabilities these cell types offer upon recellularization into acellular nerve grafts.

It was further speculated by Wüthrich et al. whether vascularization could help overcome limitations with bioengineering nerve conduits given that vascularization may promote nerve regeneration in facilitating and promoting neurotropic growth factor secretion, cell survival and growth, and provision of nutrients and oxygen (67, 68). Porcine sciatic nerves containing vascular pedicles from porcine hindlimbs were decellularized using 1% SDS and 1% Triton X-100. Upon decellularization, a patent vasculature was visualized, and nerve tissues retained most of their architecture and bundle-like structures histologically. Absence of myelinated fibers and nuclei was observed. Basal lamina of individual nerve fibers was collapsed and disorganized upon decellularization whereas the perineurium remained intact. A panel of neurotropic, inflammatory and complement factors were assessed in decellularized samples of which most were detectable and some showed reduced expression. Re-endothelialization was conducted using fluorescently-labeled wtPAEC. Following an overnight pre-conditioning of the scaffold in growth medium, wtPAEC cells were manually injected into the vasculature across four consecutive injections every half hour for 2 h total. Perfusion was started following cell injection and increased every half hour from 0.5 to 2 mL/min. Nerves were analyzed on Day 0, 1, and 7 whereby wtPAEC were observably settled in the vessels and using the endothelial marker, CD31, cells were observed closer to the pedicle and near large caliber vessels only (67). These initial results suggest that this vascularized nerve graft has biocompatibility and can support endothelial cell engraftment. However, further recellularization efforts examining recellularization from both arterial and venous conduits may be an alternative to address the lack of cells in distal parts of the pedicle. Notably, authors focused on re-endothelialization given the identified need to develop a functional vasculature to promote nerve regeneration. Co-cultures with cell types, for example vascular smooth muscle cells and pericytes, can be considered as a future step to promote vascular reconstitution.

### Composite Tissues

#### Fasciocutaneous Tissue

An engineered fasciocutaneous flap offers versatile and clinically-relevant applications in reconstructive surgery, particularly in the repair of large soft tissue defects. Additionally, the composite nature of such flaps with the epidermal cutaneous layer, the fibroblastic dermal layer, and the adipose subcutaneous layer make these tissues an attractive investigational model in VCA. To date, three reports in rat and pig models are notable for their attempts to recellularize fasciocutaneous flap tissues. The report by Chang et al. to recellularize the rat fasciocutaneous flap is one of the earliest. While the tissue flap used was not decellularized prior to recellularization the authors showed that the flap could be maintained *ex vivo* in a perfusion bioreactor and that adult stem cell populations such as bone derived and adipose derived human MSCs could be recellularized intravascularly and supported for up to 24 h *ex vivo* and up to 7 days in their transplantation studies in nude rat recipients (77).

Later work by Zhang et al. used a decellularized rat inguinal fasciocutaneous flap as an acellular scaffold for cell seeding (3). These surgically explanted free flaps were based on the superior epigastric vessels and contained skin, subcutaneous fat, and vascular tissues. Flaps were then treated with a protocol of freeze-thaw at −80 °C, and subsequently, perfused with NaCl (0.5 M/1 M), 0.25% trypsin/EDTA, isopropanol, and 1% Triton to generate acellular flaps. Recellularization relied on both vascular perfusion of HUVECs and injections of human ADSC-HUVEC co-culture with HUVECs into the dermal and subcutaneous fat layers of the flap. A similar approach of recellularizing fasciocutaneous flaps was undertaken by Jank et al. in the pig inguinal fasciocutaneous flap (7). Explanted flaps based on the superior epigastric artery were perfusion decellularized with SDS/Triton and then recellularized using 4 × 10^7^ HUVEC by gravity perfusion at 110 mmHg perfusion pressure. The recellularized flap was maintained under perfusion bioreactor conditions for five days. However, in the recellularized flaps by Zhang et al. and Jank et al., the regenerated vasculature was incomplete as evidenced by thrombosis noted after *in vivo* transplantation in both cases. In Zhang et al.*,* an occlusion of the main feeding arterial inlet was noted at 7 days post-transplantation. In Jank et al., an absence of venous outflow on gross inspection and evidence of only partial endothelial coverage only to vessel diameter of 300 *µ*m by histological examination similarly demonstrated incomplete re-vascularization in the reseeded scaffold (7). These issues of incomplete recellularization and poor post-transplant vascularization is hypothesized to be due to shear stress leading to myointimal hyperplasia and luminal occlusion or activation of the coagulation cascade caused by areas of exposed ECM created by incomplete coverage of reseeded endothelial cells. At present, the vascularization in a recellularized fasciocutaneous VCA tissue remains a key obstacle to be addressed in future work.

#### Face

Engineered facial allografts introduce added complexity to VCA tissue engineering compared to vascularized skin/adipose grafts. This is attributable to the diverse population of constituent tissues, including skin, adipose, mucosa, cartilage, and muscle. To date, recellularized facial scaffolds have been attempted with both rodent and human scaffolds.

Duisit et al. studied recellularization on full-thickness hemifacial grafts in a rodent model. An initial comparative analysis of two decellularization protocols based on either perfusion or agitation of 1% SDS detergent determined that both perfusion and agitation were equivalent in decellularization efficacy although the perfusion method was noted to have an advantage of increased decellularization of cartilage. Later seeding of rat hemifacial scaffolds was done with human ADSCs seeded statically onto 1 cm^2^ discs on both the dermal and internal surfaces and were observed to be viable using Live/Dead vital staining after 7 days of *in vitro* culture. No perfusion seeding was performed in this study (47).

In the human face, Duisit et al. utilized both segmental lower-facial and full facial allografts procured with pedicled access with the facial artery and the external jugular vein from cadaveric donors and decellularized with a protocol using sequential perfusion of 1% SDS, 1% Triton, isopropanol, and DNase. Characterization of the decellularized scaffolds demonstrated preservation of the ECM microstructure and a patent vascular tree on CT angiography. Recellularization was selectively performed on the lip subunit using both static disc and whole perfusion bioreactor formats. For static disc seeding, NIH-3T3 dermal fibroblasts and C2C12 progenitor myoblast cells were seeded atop 1 cm^2^ acellular lip discs and cultured in DMEM for 14 days. In the perfusion bioreactor, either human aortic endothelial cells (HAECs) or C2C12 myoblasts were used; HAEC were perfused into the facial artery cannula and cultured for 48 h afterwards whereas C2C12 were seeded by direct syringe injections into the lip parenchyma and cultured for 2 weeks. In both static disc and whole bioreactor lip seeding, examination of the reseeded tissue showed viable cells with evidence of attachment to the ECM. In the case of HAEC, homogenous distribution was seen in the vascular lumen with CD31 positive staining supporting the endothelial phenotype (2). While this study demonstrated encouraging results with regards to cell survival and distribution using their whole bioreactor system with an acellular lip subunit, it remains to be seen whether a perfusion bioreactor reseeding approach could be applied to the large whole face graft scaffold. Duisit et al. did not focus on functionalization of human face graft however such a composite tissue has a unique functional and anatomical tissue composition which warrants different types of recellularization strategies to mobilize different components of the face to a functional extent (2). This requires considerations for the types of tissues present in such a graft. For example, the nose and ear are cartilage-rich tissues whereas the cheeks are predominant with adipose and muscle tissues. Given the diversity of tissue composition of such a graft, recellularization of the graft as a whole may be challenging whereas targeted, partial recellularization focusing on different anatomic locations may be feasible.

#### Ear

Ear recellularization has been documented in the pig and human ear model, with both work performed by Duisit et al. (48, 50). With the porcine ear grafts, grafts were obtained from cadaveric sources and perfusion decellularized with 1% SDS and 1% Triton-X to obtain an acellular scaffold with retained ECM biochemical components and perfusable vascular tree. As with the facial scaffolds, two different formats were used for reseeding: static disc and whole bioreactor seeding. With static discs, fibroblastic Cos-7 cells and GFP-tagged MSCs were used separately used to demonstrate that the acellular scaffold could support viable and proliferating cells. For the whole-ear bioreactor seeding strategy, acellular ear scaffolds were cannulated via the artery and seeded with either MSCs or NIH-3T3 fibroblasts, each using four sequential intra-arterial injections. The system was maintained up to 2 weeks after which the recellularized ear showed homogenous distribution of cells both extra- and intra-vascularly on histology. Ear grafts seeded with MSC were also found to show engrafted cells intravascularly in both large vessels and capillaries as well as an increase in lipid content in the recellularized parenchyma using Sudan Red stain (48). Despite these encouraging results, the overall cell density in the seeded scaffold was poor, owing potentially to inadequate cell numbers for seeding.

Follow-up work in the human ear used an acellular human ear with vascular access via the external carotid artery which was decellularized with a protocol similar to the porcine ear above. Using the whole organ perfusion bioreactor system, Duisit et al. selected HAECs for intra-arterial recellularization. After *ex vivo* maturation for 48 h, intra-vascular distribution of viable HAEC was seen in both superficial and deep aspects of the tissue. However, cell coverage was inconsistent and segmented through the graft with no cells seen in the larger caliber vessels suggesting an incomplete recellularization of the human ear (50). While the recellularization of both the pig and human ear, particularly the vascular compartment, still require additional work with respect to cell coverage and engraftment, recellularization of ear scaffolds is a technological advancement that has brought attention to whole-tissue reseeding as a promising strategy in VCA engineering.

#### Larynx

The larynx is a complex composite tissue that is composed of various components including muscle, cartilage and mucosa. The first tissue engineered larynx study can be attributed to Hou et al. using a decellularized rabbit larynx with SDS/Triton-X. Subsequent *in vitro* whole-scaffold recellularization with MSCs and myogenesis-induced MSCs demonstrated the formation of a muscle-like structure after 8 weeks of heterotopic transplantation within recipient rabbit omentum (78).

A more recent study by Moser et al. attempted canine larynx recellularization with multiple cell populations (8). Procured larynxes were decellularized and recellularized all within a custom-made perfusion bioreactor. Notable in this study is their strategy of recellularization using three distinct cell populations: human airway basal cells, primary human myoblasts, and HUVECs. To facilitate their approach, the investigators validated a tailored culture media, termed “multi-cell growth medium,” that permitted concurrent culture of all three cell populations. Additional experiments with whole larynx HUVEC cell seeding supported evidence of endothelialization to different vascular calibers after perfusion culture of the scaffold for three days (8). In both the two mentioned cases of Hou et al., and Moser et al. however*,* no orthotopic transplantation of the larynx was performed thus leaving open the question of whether functional recapitulation of a tissue engineered larynx is achievable.

#### Trachea

A tissue engineered trachea has received much attention as a clinically viable solution in long-segment tracheal replacement. However, attempts to regenerate a functional trachea using naturally derived scaffolds and exogenous cells have seen mixed success and recent clinical studies have cast doubt on its feasibility (70). Nonetheless, work with trachea regeneration offers pertinent insights that can be applied to multi-tissue recellularization needed in VCA engineering. Using the pig as a clinically relevant animal model, Haykal et al. applied a novel double chamber perfusion bioreactor to seed long-segment decellularized tracheal scaffolds with separate luminal and external compartments for seeding of tracheal epithelial cells and bone marrow-derived MSCs, respectively. Over an *ex vivo* maturation period of 72 h, recellularization of the trachea within this double-chamber perfusion bioreactor showed improved cell attachment and distribution compared to traditional static-seeding methods without perfusion (9). Additional advancement of porcine trachea regeneration by Aoki et al. described a chimeric trachea recellularization approach characterized by partial decellularization of the donor tracheal epithelium while preserving an immune-privileged cartilage compartment (10). Subsequent re-epithelialization of the trachea scaffold using exogenous human bronchial epithelial cells matured in an *ex vivo* perfusion bioreactor over 7 days facilitated cell attachment, viability and growth. Altogether, these two descriptions of tracheal engineering that utilize dynamic perfusion seeding within a double chamber bioreactor and a recellularization approach based on donor-recipient tissue chimerism are potential concepts that may be adopted to the broader scope of recellularization of other complex VCA tissues.

#### Limb

Jank et al. conducted successful perfusion decellularization of a rat forelimb and primate forelimb using 1% SDS and 1% Triton X-100. Preservation of vessels, nerve, bone, muscle, and skin was observed histologically. Biomechanical properties of the composite forelimb scaffold showed decellularization did not affect joint flexibility nor mechanical, geometric, and mineral properties of the bone. Perfusion recellularization was conducted in several phases using co-cultures and tri-cultures of HUVECs, C2C12 mouse myoblasts, and mouse embryonic fibroblasts to accommodate regeneration of different tissue compartments. The grafts were perfused and matured in a biomimetic bioreactor system with electrical stimulation for 21 days total. Perfusion and injection seeding strategies were tested separately with myoblasts. Perfusion seeding resulted in marginal distribution of myoblasts in the muscle ECM with most cells retained in the vasculature whereas injection seeding allowed cell engraftment and formation of muscle-like tissue. While this allowed targeted cell delivery, it also resulted in apoptosis at injection sites and disruption to the ECM. This highlights important considerations in selecting appropriate seeding strategies for recellularizing a physiologically dense tissue such as skeletal muscle. Functional testing using isometric force contraction of recellularized muscle showed some tetanic contractile force response relative to native. Upon orthotopic limb transplantation using recellularized forelimbs, the arterial network filled with blood however venous return was not reported. Flexion was observed at the wrist and metacarpophalangeal joints upon electrical stimulation of the transplanted recellularized grafts. Electrical stimulation can evidently allow enhanced myofiber alignment and muscle maturation and while authors suggest electrical stimulation enhanced myofiber alignment in the endomysium during cell growth and differentiation phases, recellularization without electrical stimulation was not conducted to show the extent of muscle regeneration with or without it (51, 65).

#### Genitourinary

Penile tissues are a recent addition to VCA tissue engineering and present unique compositions and functional needs for regeneration. The penile scaffold features corpora cavernosa required for erectile function, spongy corpus spongiosum tissue which surrounds the urethra, and additional fibrous and fascial layers surrounding these structures along with a skin/adipose tissue enveloped exteriorly. This penile graft houses heterogenous and unique tissue compartments.

Tan et al. pursued perfusion decellularization and recellularization of human penile scaffolds. Decellularization was conducted in a hybrid fashion via micro-arterial perfusion and urethral perfusion using 1% SDS with slow mechanical agitation simultaneously. An intact tissue ECM architecture was maintained post-decellularization along with low residual DNA levels (less than 50 ng/mg), maintenance of collagen, laminin, fibronectin, and growth factors: VEGF, endothelial growth factor, and transforming growth factor beta-1. A perfusable vascular tree was also observed. Human adipose-derived stromal vascular fraction (SVF) cells were statically seeded on sectioned slices of the decellularized scaffolds and cultured for 28 days. Cells showed adherence, viability, and proliferative capabilities in the cavernosa, tunica, and urethra. Cells showed lining along luminal structures in the cavernosa as well (53). Evidence of early formation of vascular microtubes and increased expression of endothelial marker, von Willebrand factor, in the reseeded cavernosa after 28 days of *in vitro* scaffold culture was reported (53). Derived from discarded human-derived adipose tissues, the SVF contains a heterogenous cell combination such as adipose derived stem cells (ADSCs), endothelial primary cells, endothelial progenitor cells, immune cells, smooth muscle cells, and pericytes. The varied cell composition of SVF have been shown to exhibit beneficial immunomodulatory and angiogenic properties, which can contribute to their regenerative and therapeutic potential (79, 80). Thus far, Tan et al.’s study is the only study that has used SVF for recellularization.

While Tan et al. focused on the composite scaffold, some authors have previously focused on tissue-engineering corpora cavernosum tissue given the lack of a suitable substitute for penile reconstruction (53, 81). Ji et al. evaluated the use of muscle-derived stem cells for recellularizing acellular corpora cavernosa matrices. Tissues were obtained from male rabbit penises and subjected to decellularization with 2% Triton X-100 and 0.1% ammonium hydroxide. Tissues were simultaneously agitated and decellularized for 10 days. Tissue structure and organization was maintained post-decellularization although cavernosal sinus lacunae were irregular and larger post-decellularization relative to native tissue. Muscle-derived stem cells were isolated and statically seeded for recellularization. The cells became aggregated in the corpus cavernosum 5 days after recellularization. While cell proliferation was observed, some cell death was noted. Authors used the expression of α-smooth muscle actin (α-SMA) as an indication for muscle-derived stem cell differentiation. Decellularized tissues were also transplanted in normal rabbits and evaluated 2-, 4-, and 6-months post-transplantation. Neovascularization was noted at every timepoint. After 4 months, cell proliferation and cell distribution was observed however no differences could be observed after 6 months (81). Ji et al.’s study was extended by An et al. where authors used overexpression of VEGF to enhance contractility of recellularized corpora cavernosa tissues which were obtained using the same protocol (71). Muscle-derived stem cells were transfected with VEGF lentiviral gene vector and seeded onto decellularized cavernosa tissues. VEGF-expressing cells elucidated better growth and engraftment relative to non-transfected cells. α-SMA for cell differentiation and endothelial markers, CD31 and vWF, were increased in scaffolds recellularized with VEGF-expressing cells (71). Along with establishing successful de- and recellularization methods for the corpus cavernosum, these authors also highlighted the potential use of growth factor supplementation to enhance recellularization.

While the urethra has not been discussed for VCA, it is worth consideration given its vascularity and unique dual blood supply that allows it to be mobilized and divided to construct a fasciocutaneous onlay flap for urethral reconstruction (72). Further, it contains an internal mucosal layer, a middle smooth muscle layer, and an external urethral sphincter comprised of skeletal muscle myofibers, altogether introducing tissue complexity that requires customized recellularization approaches.

Simões et al. conducted the first perfusion decellularization and recellularization study of whole porcine urethras. Authors identified that there is a lack of whole urethral decellularization efficiency which hinders the ability to bioengineer physiologically relevant urethral tissues. Porcine urethras were cannulated at their most distal region and perfused with 0.5% and 1% SDS. Cell removal was observed and ECM characterization showed retention of various proteins albeit with a noted ∼50% reduction in elastin content. For recellularization, cross-sections of acellular urethra scaffolds containing the mucosa, smooth and skeletal muscle ECM were produced using a cryostat and conditioned in culture medium prior to cell seeding. Static seeding was performed using highly concentrated cell suspensions of the following cell types: human muscle progenitor cells, human bone marrow MSCs, L929 fibroblasts, and SVF. All cell types were seeded separately in individual experiments and were selected due to their role in muscle and sphincter formation, and tissue regeneration. Bioscaffolds showed cell engraftment, viability, and proliferation up to 2 weeks across all cell types. Muscle progenitor cells showed fusion and ability to form multinucleated fibers, expressing markers of skeletal muscle. Spontaneous contraction could also be observed upon myogenic induction (52). While these recellularization studies were conducted *in vitro,* they substantiate the potential of regenerating different components of the urethral tissue that can help support regeneration of larger tissues.

The uterus comprises of an endometrium with endothelial cell lining, surrounded by muscle-cell rich myometrium and a perimetrium comprising of epithelial cells. Miyazaki & Maruyama conducted decellularization of rat uteri via aortic perfusion using an SDS protocol from previous liver decellularization studies involving progressively increasing SDS concentrations from 0.1%–1%. A perfusable and maintained tissue ECM architecture was observed. For recellularization, authors injected a combination of primary neonatal and adult uterine cells, and rat MSCs throughout the uterine wall. Collagen gel was used to cover the graft post-injections to prevent cell leakage. The graft was then perfused in a bioreactor system. Endometrial-like tissue formation was observed and partial cellular distribution in the matrix by Day 3. Vessel-like structures positive for CD31 and smooth muscle actin were observed. The graft became increasing atrophic by Day 6 however was sustained until Day 10 (82).

Campo et al. extended uterine decellularization and recellularization to a porcine model, using both fresh and freeze-thawed uteri. Decellularization entailed comparison of two SDS and Triton X-100 protocols whereby no difference was found in terms of ECM characterization and vascular patency. Acellular disk scaffolds were created by taking punch biopsies of the decellularized uteri tissue, with the endometrial tissue facing upwards. Human endometrial stem cells were statically seeded and cultured for a total of 12 days under hypoxic conditions. Contraction of the recellularized tissue was observed 3–4 days post-seeding. The recellularized disks arranged into 3D structures 9–12 days post-seeding. Endometrial intracellular proteins, vimentin and cytokeratin, were detected (49).

Upscaling into an ovine model, Tiemann et al. employed a sodium-deoxycholate (SDC) detergent protocol for decellularization of ovine uteri after trialing three different decellularization protocols. Vascular patency and ECM architectural maintenance was observably superior in the SDC approach. Interestingly, authors froze isolated uteri at −20 °C before beginning recellularization. Rings of the decellularized tissue were made using a cryostat and recellularized using sheep fetal bone marrow stem cells. Cells were injected into the ring of tissue, either with 1 × 10^6^ or 1 × 10^7^ cells. Rings were cultured until Day 3 and Day 14. Regardless of seeding density, cells remained in the superficial layers, at the injection site. Cells showed expression for proliferation marker, Ki67, and with additional endometrial surface protein markers. No differences were seen in expression between Day 3 and Day 14 (55). Despite the lack of cell migration into deeper parts of the ovine tissue, cells remained viable for a two-week period suggesting a non-toxic culturing environment. Further, the use of an ovine model is evidently suitable for extrapolating translatable findings as the uteri anatomy and size is similar to that of humans relative to cow and porcine uteri.

## Challenges in Composite Tissues Recellularization

### Functional Regeneration

Successful recellularization is dependent on the tissues of interest and the effect of recellularization strategies on cellular properties, the ECM, vascular integrity, and functionality of scaffolds. One of the most significant challenges in recellularization is regenerating tissues to functional capacities, with added difficulty in recellularizing composite grafts. Understanding the functional requirements of each tissue in a composite model is required to dictate and personalize recellularization strategies for respective tissues. The structural differences across tissue compartments such as tissue density, the anatomic location of tissues, and the tissue architectures are all variables to consider. Dense tissues may require alternative cell seeding strategies such as perfusion seeding through the native vasculature, injections to target physiologically dense tissues, and/or biophysical stimuli to promote cellular differentiation and maturation. Location of tissues in a composite model also dictates functionality given the exposure grafts receive during both decellularization and recellularization. Questioning what respective tissues require for functionality and how to mimic such behavior in recellularization is required to model and evaluate recellularization efforts for regaining tissue function.

Assessing recellularized tissue morphology and structural integrity can elucidate the potential for functional restoration, using techniques such as standard electron microscopy (SEM). SEM of porcine fasciocutaneous groin flap, rat skin/adipose flap, porcine and human ear grafts, human penile tissue, porcine urethra, porcine and ovine uteri all reported preservation of tissue ultrastructure, with a porous morphology observed post-cell removal (3, 7, 47–49, 52, 53, 55). However, transmission electron microscopy (TEM) would be a beneficial addition to such studies, to provide a deeper view of tissue ultrastructure.

### Nerve Regeneration

Nerve regeneration has also been a longstanding challenge in VCA tissue recellularization approaches. Tissue reperfusion is not sufficient without restoration of functional innervation, particularly for the muscle and the skin. For VCA grafts such as the face and the limb, nerve regeneration is critical to modulate motor and sensory function along with helping functionalize other tissues dependent on innervation, such as the muscle. However, nerve regeneration is slow, and dependent on its surrounding microenvironment and nerve caliber (73).

Axonal regrowth is reliant on growth factors to support nerve regeneration. Notable examples include brain-derived neurotropic factor, nerve growth factor, and VEGF which have been previously employed and deemed to be promoters of axonal regrowth (67, 83). Wüthrich et al. in particular assessed the preservation several growth factors post-decellularization whereby detectable levels could be observed. While they did not focus on characterizing this profile of growth factors, their preliminary work elucidated that some of the key growth factors remain post-decellularization and could be beneficial in promoting nerve cell growth (67). Additionally, the interplay of the ECM with nerve function cannot be missed where components such as GAGs have known interactions with neurotropic factors such as promoting neural adhesion and migration (67, 84). This necessitates evaluation of the ECM components such as GAGs post-decellularization to corroborate its potential role in influencing nerve regeneration. Further. the complex ECM milieu has various interactions with cell functions and tissue structures to facilitate nerve signal conduction. Determining the functional capacity of regenerated tissues therefore requires a concerted investigation across ECM constituents and their relative presence, level of tissue functionality, and capability of cellular activities such as proliferation and differentiation.

Considerations for the use of electrophysiology to mimic neuronal activity during recellularization is also pertinent and often relies on functional analyses of muscle tissue in composite grafts. Provision of electrical stimulation during recellularization can replicate innervation required by muscles to stimulate and enact contractions. Nerve regeneration can be evaluated using histological methods such as toluidine blue staining, immunostaining for neurofilaments, conducting transmission electron microscopy and electrophysiology. Retrograde labeling, also used for evaluating nerve regeneration, is beneficial in functional analyses as it helps correlate the role of cells used in recellularization and their potential impact on tissue function (74).

### Vascular Regeneration

The presence of a vasculature is especially important to cells that compose a substantial portion of composite allografts include metabolically active cells that rely on a robust blood supply for growth and support. Furthermore, a patent and functional vascular architecture is a central tenet to vascularized composite tissues during reconstructive surgery. In the case of nerve regeneration, vascularization provides nutrients and oxygen to support nerve regrowth (68). Cell survival and integration has also been evidently maintained in various tissue engineered scaffolds by including a vascularization component. Wüthrich et al. investigated the potential benefit of including functional vascularization in the decellularized porcine sciatic nerve constructs, given its known effects on promoting Schwann cell migration, cell growth and survival, and in secreting neurotropic factors critical for nerve regeneration (67, 68). Until resolved, the problem of vascularization represents a significant barrier to the long-term success of an engineered allograft and its translation from lab to the clinic.

The examination of the vasculature is critically important to regenerating a functional and perfusable vascular network in vascularized composite tissues. Non-invasive, non-destructive method to assess the recellularized vasculature can assist characterization and visualization. Assessments of vascular integrity includes the use of microparticles, to assess for endothelial leakage or extravasation. As previously reported in the recellularized lung, perfusable microspheres (0.2 *µ*m diameter) can be used to examine an acellular lung scaffold vasculature and demonstrate areas of preserved integrity and continuity (37, 85). Another strategy that can be employed to evaluate vascular integrity is the use of tagged biological markers such as Evans-Blue labelled BSA or FITC-labelled dextran as reported previously (13, 86). In the former case, the use of Evans Blue conjugated with BSA has been shown to help identify areas of incompletely recellularized endothelium (26).

Radiographic techniques such as the use of fluorescent dyes (e.g., Indocyanine Green) or radioopaque contrast agents (e.g., Microfil/µAngiofil) can also provide another perspective of reengineered tissue perfusability (87). The use of microtomography and microCT imaging with intra-vascular Microfil has been described in previous recellularization attempts with the inguinal fasciocutaneous flap whereas a decellularized vascularized nerve scaffold could be imaged down to the capillary level using the µAngiofil contrast agent (3, 7, 67). Another imaging technique with potential application in evaluating VCA vascularization is real-time imaging (e.g*.*, laser doppler perfusion imaging) that allows for serial observations over time without graft sacrifice (88). Ultimately, the application of this broad diversity of techniques to VCA tissue engineering would greatly benefit the field to increase the completeness of the vascularity in tissue regeneration.

## Future Directions

On a cellular level, future work should better define the appropriate cell characteristics that are needed to adequately recellularize tissues. With a view towards clinical scalability, the cell numbers needed for full graft recellularization is not completely determined and varies according to tissue type and composition. The unique cell populations to recapitulate the various tissue niches in VCA grafts also requires further consideration. For example, different cell phenotypes will be needed to repopulate the vascular versus the parenchymal compartment, each needing individualized growth and maturation conditions. While the use of endothelial cells has been the most prevalent, no study has examined the combined role of mural cells for recellularization in VCA. Work by Ren et al. in whole organ engineering of the rat lung demonstrated improved endothelial barrier function and decreased vascular resistances using ECs and pericytes co-seeded onto the acellular lung matrix (37). These encouraging results can potentially be extrapolated to VCA tissues by pre-seeding scaffolds with pericytes and/or vascular smooth muscle cells to augment the attachment and survival of ECs, a strategy also suggested by previous authors (12, 67). Future work is also needed to examine short-term versus long-term performance of cells in engineered tissues. To this end, additional cell-evaluation techniques and platforms need to be developed can be used monitor the course of cell maturation and differentiation, ideally in real-time. Real-time monitoring of cells, particularly for those derived from multipotent stem cells will be important to mitigate the potential of deranged cell differentiation causing tumorigenesis. Finally, evaluation of scaffolds for evidence of cytotoxicity will be important for ensuring engineered tissues will be safe for clinical translation.

A composite population of both luminal endothelial and circumferential support cells (i.e., vascular smooth muscle cells and pericytes) will be needed to support the integrity and long-term functionality of a regenerated vascular system. Further, more work with stem cells is also needed to ensure that differentiation is appropriate to maintain correct tissue tropism while avoiding the possibility of tumorigenesis arising from deranged differentiation (89). Applying the use of newly emerging –omics technologies will assist in improving our current engineering strategy with a greater understanding of cellular interactions at the molecular level. Next generation techniques such as single-cell RNA sequencing of bioengineered tissues has attempted with notable examples published recently in lung regeneration that utilize single cell RNAseq to assess alveolar epithelial and endothelial regeneration in recellularized whole lung scaffolds (90, 91). However, to date, such applications of novel technologies of single-cell analyses such as transcriptomics, proteomics, epigenomics, and multiomics studies have yet to be fully applied within the VCA context and thus represent a substantial and exciting open area of future research.

### Technical Factors

Beyond cellular biology, future work will also be required to determine the appropriate cell seeding methodology. As reviewed, a multitude of recellularization techniques have been reported in literature. Given the various different tissue compartments in VCA tissues, a multimodal recellularization approach is potentially needed to recapitulate the diverse cell populations found in native tissues. One hypothesized approach is sequential seeding using staged cellularization, for example seeding with a basal supporting cell type first followed next by a luminal cell layer (i.e., endothelial or airway epithelial cell). In the case of the vascular compartment of VCA, the avoidance of thrombogenesis in the regenerated tissue is especially important to ensure a perfusable and functional bioartificial graft. Unfortunately, current approaches to reconstruct a physiologically functional endothelium without thrombosis have been unsuccessful. Potential solutions for future investigation can include the covering residual exposed ECM areas with cross-linking of different antithrombotic chemicals and agents (26). The extent to which these approaches can reduce thrombogenicity and prevent long-term graft failure *in vivo* remains to be determined.

Moreover, additional study of recellularization dynamics should be directed to understanding the biophysical parameters that influence cell distribution during the recellularization process. Such strategies can include spatiotemporal computational modeling of cell distribution into grafts in the setting of varying physical parameters such as cell number, flow rate and recellularization access. Computational models have been attempted in whole organ constructs (92, 93) although no such study has been performed in the VCA setting. These studies will permit more in-depth analysis of the recellularization process. Another concept for future consideration is whether VCA grafts following recellularization will require full maturation *ex vivo* or whether a tandem approach that combines an initial *ex vivo* bioreactor recellularization phase followed by completion of full graft maturation *in vivo* would be superior.

Operational and engineering improvements will also be needed to advance future VCA regeneration efforts. One area in need of more development is in bioreactor technology that is compatible with regenerating advanced composite tissues. Future bioreactors can incorporate added functionalities such as real-time monitoring or permit increased scalability with greater throughput and turn-around time. To this end, large GMP facilities will need to be developed that can expand the recellularization cells in a standardized and cost-efficient method. Time-sensitivity of tissue regeneration will be important for patients that require regenerated tissues and organ on an urgent or acute basis.

Also pertinent to VCA regeneration is an elaboration of specific design criteria to define appropriately regenerated VCA grafts. These criteria will allow greater operational efficiencies and standardization to the VCA tissue engineering process. Important parameters to consider include biological characteristics such as cell phenotype, graft biocompatibility, and post-transplantation immune-tolerance in recipients. As the use of *ex vivo* bioreactors requires tailored physiological environments to regenerate tissues, stringent design and operational considerations over the course of tissue regeneration are also important. These can include bioreactor process and quality control measures, bioreactor sterility maintenance, and workflow reproducibility.

### Clinical Translatability

Finally, with a view towards ultimate translation into patient care, a few clinical considerations should be made. First, additional studies incorporating *in vivo* transplantation of reengineered tissue grafts will be needed to determine the biocompatibility of these grafts, ideally with pre-clinical studies in animals. Biocompatible engineered VCA tissues will need to fulfill several conditions before such constructs can be safely used in human patients. One such condition is immunotolerance of engineered tissues in a human recipient following transplantation. While it is hypothesized that use of recellularized acellular scaffolds in VCA could potentially mitigate immunosuppression requirements, it remains to be determined whether engineered tissues are truly immunologically inert. When using decellularized animal tissues as a scaffold source, future work will need to ensure that such scaffolds do not possess xeno-antigens than can potentially elicit a hyper-acute rejection and cause graft failure in the recipient. Secondly, future pre-clinical and clinical trials will also be needed to establish the clinical efficacy and safety profile by comparing tissue engineered VCA constructs to conventional care standards in reconstructive surgery. This translational research will be critical to integrate this innovative technology into clinical practice in the future.

## Conclusions

VCA tissue engineering is a nascent field with enormous potential for substantial scientific and clinical impact to reconstructive surgery. Hypothetically, an engineered VCA graft could permit the *de novo* regeneration of VCA tissues without incurring the risks of complications arising from donor site morbidity (e.g*.*, wound infections, iatrogenic injury, *etc.*) in the case of autologous grafts. Furthermore, tissue engineered grafts is a promising solution to address clinically relevant problems in VCA such as allograft immunogenicity and donor availability. The strategy to use recellularized scaffolds is preferential as it relies on a microarchitectural and structural framework that is more biomimetic compared to alternative tissue engineering methods such as three-dimensional tissue printing or synthetic scaffolds (4, 94). For successful VCA bioengineering, a convergence of several components is required, including defined cell population(s), a biocompatible ECM scaffold, and an *ex vivo* bioreactor for functional maturation. As reviewed, various groups have contributed to the field of VCA recellularization, each providing different approaches to achieve an overall objective of regenerating viable and functional tissues. Current VCA strategies for de- and recellularization involve an added *in vitro* and *ex vivo* step to modify the donor biological identity into the recipient’s. While much work has been done to pioneer the approach to regenerating a VCA allograft using a decellularized tissue scaffold, the next frontier of recellularization requires much effort before such a strategy is clinically viable and translatable. Looking ahead, the future of VCA tissue engineering will require a concerted, multidisciplinary effort to better characterize the recellularization process and regenerate tissues with greater functionality.
